# Prostate cancer screening among elderly men in Brazil: should we diagnose or not?

**DOI:** 10.1590/S1677-5538.IBJU.2019.0022

**Published:** 2020-01-13

**Authors:** Rafael Ribeiro Mori, Eliney Ferreira Faria, Edmundo Carvalho Mauad, Antonio Antunes Rodrigues, Rodolfo Borges dos Reis

**Affiliations:** 1 Faculdade de Medicina de Ribeirão Preto, Universidade de São Paulo, Ribeirão Preto, SP, Brasil; 2 Hospital de Câncer de Barretos, Barretos, SP, Brasil

**Keywords:** Prostatic Neoplasms, Early Detection of Cancer, Diagnosis

## Abstract

**Purpose:**

Prostate cancer screening in the elderly is controversial. The Brazilian government and the National Cancer Institute (INCA) do not recommend systematic screening. Our purpose was to assess prevalence and aggressiveness of prostate cancer in men aged 70 years and above, on the first Latin American database to date.

**Materials and Methods:**

Cross-sectional study (n=17,571) from 231 municipalities, visited by Mobile Cancer Prevention Units of a prostate-specific antigen (PSA) based opportunistic screening program, between 2004 and 2007. The criteria for biopsy were: PSA>4.0ng/ml, or PSA 2.5-4.0ng/ml with free/total PSA ratio ≤15%, or suspicious digital rectal examination findings. The screened men were stratified in two age groups (45-69 years, and ≥70 years). These groups were compared regarding prostate cancer prevalence and aggressiveness criteria (PSA, Gleason score from biopsy and TNM staging).

**Results:**

The prevalence of prostate cancer found was 3.7%. When compared to men aged 45-69 years, individuals aged 70 years and above presented cancer prevalence about three times higher (prevalence ratio 2.9, p<0.01), and greater likelihood to present PSA level above 10.0ng/ml at diagnosis (odds ratio 2.63, p<0.01). The group of elderly men also presented prevalence of histologically aggressive disease (Gleason 8-10) 3.6 times higher (p<0.01), and 5-fold greater prevalence of metastases (PR 4.95, p<0.05).

**Conclusions:**

Prostate cancer screening in men aged over 70 may be relevant in Brazil, considering the absence of systematic screening, higher prevalence and higher probability of high-risk disease found in this age range of the population studied.

## INTRODUCTION

Prostate cancer (PCa) is a major issue in cancer incidence and mortality worldwide (1). In the United States of America (USA), the Surveillance, Epidemiology and End Results Program (SEER) data estimated 161.360 new cases of PCa in 2017 (incidence rate 119.8/100.000) (2). PCa is the most commonly diagnosed non-skin neoplasm in American males, and also the third leading cause of cancer death in men (3).

Despite that numbers, the effects of prostate-specific antigen (PSA) screening on mortality are controversial. The two large prospective randomized trials showed conflicting results. The European Randomized Study of Screening for Prostate Cancer (ERSPC) found a 27% relative reduction in PCa mortality in the intervention arm (4). On the other hand, the Prostate, Lung, Colorectal and Ovarian cancer screening study (PLCO) found no benefits (5). Recent analyses on these data reported PLCO methodological flaws, dismissing its capability of evaluating systematic screening effectiveness. The current US Prevention Services Task Forces draft recommendation states that screening reduces mortality, in spite of harms associated with overdiagnosis and overtreatment (6, 7). There is consensus among medical entities about performing PCa screening only in individuals with life expectancy superior to 10 years (7–9), due to the long cancer-specific survival after the diagnosis of localized disease (10).

Although there are recommendations against PCa screening over the age of 70 (7, 8), some epidemiologic and biologic phenomena justify the investigation of PCa assessment in this population group. First, the age shift: many authors reported demographic changes observed in the last decades in developed countries, where the increment in longevity leads to increasing incidence of senescence-related diseases, like PCa (11). Second, PCa epidemiology: this neoplasm still predominates in the elderly, although PSA screening increased the detection of PCa in younger men. In USA, 57.4% of the new diagnoses were made in men aged ≥65 (2). The literature is still scarce concerning screening in this specific age range. ERSPC (4) and PLCO (5) included few men aged ≥70, and none ≥75. Third, the disease seems to present more unfavorable features when diagnosed in older men: Brassell et al. reported that the clinical and pathological characteristics of PCa worsen with ageing, and men aged ≥70 had worse overall- and cancer-specific survival when compared to younger men (12).

In Brazil, PCa is also a public health problem (13), but data assessing screening are limited, and underprivileged people have difficult access to specialized healthcare system. Unlike many developed countries, systematic screening (defined by PSA testing, with or without digital rectal examination, on a time-regular and age-interval defined basis) has never been performed in the Brazilian population (14). It is important to highlight that, although Brazil follows the trends of age shift (13, 15), the government and the National Cancer Institute (INCA) recommend against systematic PCa screening (16), in opposition to the Brazilian Society of Urology (SBU) favorable recommendations. Considering that scenario, we hypothesized that individuals aged over 70 years and not previously screened may present more aggressive disease at diagnosis.

## OBJECTIVES

To test the hypothesis that elderly men (aged 70 and above) of a Brazilian population not exposed to previous systematic screening have increased prevalence of prostate cancer and worse aggressiveness criteria at diagnosis, compared to younger men.

## MATERIALS AND METHODS

We performed an analysis on the database of the Mobile Cancer Prevention Units (MCPU) program (14), which visited 231 cities of six Brazilians states between 2004 and 2007, including 17.571 volunteers for PSA-based PCa screening, aged ≥45 years. There was no upper age cut-off on those willing to volunteer, as reported by Faria et al. (14, 17, 18).

The criteria for prostate biopsy were: suspicious digital rectal examination (DRE) findings, or PSA >4.0ng/ml, or PSA 2.5-4.0ng/ml with free/total PSA ratio ≤15% (which was performed to assess the increase in the PCa detection rate in one preliminary study of the MCPU program, when the literature lacked data to the date) (18).

The initial biopsy protocol consisted of transrectal ultrasound (TRUS) guided sextant biopsies. The protocol was changed in November 2004 to mean 14 cores (eventually with additional samples of suspicious areas on TRUS or DRE).

Men with a positive biopsy were clinically staged by magnetic resonance imaging and conventional bone scan. The TNM staging (6^th^ ed.), Gleason score histologic classification (score 2-10 to date) and D'Amico risk stratification systems were utilized.

All men with Gleason score 2-6 (to the date) were considered in the same category “Gleason 2-6” in our study, which currently corresponds to the International Society of Urologic Pathology - ISUP grade group 1 (19).

The whole health care, from screening to treatment, was provided through the public health system at the Barretos Cancer Hospital (BCH).

### Study design

A cross-sectional study was performed on the database. The screened men were stratified in two age groups (45-69 years, and ≥70 years). These groups were compared regarding PCa prevalence, previous PSA testing and aggressiveness criteria like PSA levels, Gleason score from biopsy and clinical TNM staging, as well as other relevant variables. The present study protocol was reviewed and approved by the ethics committee of the Hospital das Clinicas of the Ribeirao Preto Medical School of University of Sao Paulo (n. 1.639.824).

## Statistical analysis

We used the Statistical Package for Social Sciences software version 17.0 (SPSS, Chicago, IL) for the statistical analysis, using χ^2^ (chi-square) tests and Bonferroni correction. According to the study's cross-sectional design, the preferential measure of association utilized was the prevalence ratio (PR). When appropriate, we also calculated the odds ratio (OR). The significance level considered was p <0.05 for all tests.

## RESULTS

### Age, PSA levels and previous PSA testing

The population studied included 17.571 men with median age of 60 years (range 45-98) and mean PSA level of 2.0ng/ml [SD 6.1, CI95% (1.9-2.1)], 5.108 individuals (29.1%) had at least one previous PSA testing. The median age of men diagnosed with PCa was 68 years.

Group A (age 45-69) enrolled 14.287 men, with median age of 58 years (63 years in men with PCa), mean PSA level of 1.6ng/ml [SD 3.5, CI95% (1.52-1.64)] and 28.2% of men with previous PSA testing.

Group B (age ≥70) accounted 3.284 men, with median age of 74 years (the same in men with PCa of this group), mean PSA level of 3.9ng/ml [SD 11.8, CI95% (3.5-4.3)] and 32.7% of previous PSA testing.

The prevalence of previous screening was 15.7% higher in men aged ≥70 [PR 1.157, CI95% (1.1-1.2), p <0.05], but there was no difference when we compared only men with positive biopsies [PR 1.2, CI95% (0.9-1.5), p=0.11].

### PSA levels and PSA range stratification in men with positive biopsies

Considering only PCa cases, the mean PSA level in our sample was 12.7ng/ml [SD 23.8, CI95% (10.9-14.6)]. The mean PSA levels were higher in the group of men aged ≥70: the mean PSA level of group A was 9.5ng/ml [SD 13.2, CI95% (8.2-10.9)], while mean PSA level of group B was 17.3ng/ml [SD 33.0, CI95% (13.3-21.2)].

PSA levels were in higher ranges in the group of elderly men, when compared to the group of age 45-69: PCa cases in the group of elderly men were 92% more likely to present with PSA levels within the 4-10ng/ml range [OR 1.92, CI95% (1.3-2.9), p <0.01], and 2.6 times more likely of being diagnosed within the PSA range above 10ng/ml [OR 2.63, CI95% (1.7-4.0), p <0.01].

### Prostate biopsy results and prostate cancer prevalence

Two thousand eight hundred and forty-one men were called for specialized evaluation at the BCH (16.2% of the sample). 2.291 individuals showed up (80.6% of the invited) with 1.647 men biopsied (58% of the invited). The criterion “PSA >4ng/ml” accounted for 51% of the biopsies performed. The distribution of the other criteria was: abnormal DRE (19.7%), both altered PSA and DRE (7.1%), and free/total PSA ≤15% (18.3%).

The overall prevalence of PCa in our study was 3.7%, and the biopsy positivity rate was 39.6%, as shown in Table-1. The group of men aged ≥70 presented a prevalence of PCa three times higher than those aged 45-69 [PR 2.9, CI95% (2.5-3.4), p<0.05].

**Table 1:** Prostate biopsy results and prostate cancer prevalence.

	Total	Group A (age 45-69)	Group B (age ≥70)	PR	p	CI95%
**Biopsied men**, n (prevalence)	1,647 (9.4%)	1,088 (7.6%)	559 (17%)	2.24	<0.01	(2.0-2.5)
**PCa cases**, n (prevalence)	652 (3.7%)	382 (2.7%)	270 (8.2%)	2.9	<0.01	(2.5-3.4)
**Biopsy positivity rate**	39.6%	35.1%	48.3%	1.38	<0.01	(1.2-1.5)
**Total, n**	**17,571**	**14,287**	**3,284**	**-**	**-**	**-**

Abbreviations: **PR** = prevalence ratio; p = p value; **CI95% =** confidence interval 95%; **n** = number of men; **PCa** = prostate cancer

### Gleason score

The prevalence of histologically aggressive disease (Gleason score 8-10) was higher in the group of elderly men [PR 3.6, CI95% (1.9-6.7), p <0.01], as demonstrated in Table-2.

**Table 2:** Gleason score from biopsy.

	Total	Group A (age 45-69)	Group B (age ≥70)	PR	p	CI95%
**Gleason 2-6**, n (%)	440 (67.5%)	276 (72.2%)	164 (60.8%)	0.8	<0.01	(0.7-0.9)
**Gleason 7**, n (%)	166 (25.5%)	93 (24.4%)	73 (27%)	1.1	0.06	(0.8-1.4)
**Gleason 8-10**, n (%)	46 (7%)	13 (3.4%)	33 (12.2%)	3.6	<0.01	(1.9-6.7)

Abbreviations: **PR =** prevalence ratio; **p =** p value; **CI95% =** confidence interval 95%; **n** = number of men

### TNM staging

In our sample, 93.4% of men with PCa presented with localized disease (T1/T2). There was no significative difference between the groups, regarding T2-T4 staging and lymph node staging, as shown in Table-3. Metastatic disease was almost five times more prevalent in men aged above 70 years [PR 4.9, CI95% (1.6-14.9), p <0.05].

**Table 3:** TNM Staging.

	Total	Group A (age 45-69)	Group B (age ≥70)	PR	p	CI95%
**cT**						
**T1**, n (%)	498 (76.4%)	305 (79.8%)	193 (71.5%)	0.9	<0.05	(0.82-0.98)
**T2**, n (%)	111 (17.0%)	56 (14.7%)	55 (20.4%)	1.39	0.09	(0.99-1.9)
**T3**, n (%)	40 (6.1%)	20 (5.2%)	20 (7.4%)	1.42	0.09	(0.8-2.6)
**T4**, n (%)	3 (0.5%)	1 (0.3%)	2 (0.7%)	2.83	0.09	(0.3-31.1)
**cN**						
**N0**, n (%)	624 (95.7%)	371 (97.1%)	253 (93.7%)	0.96	0.05 (NS)	(0.9-1.0)
**N1**, n (%)	26 (4.0%)	11 (2.9%)	15 (5.6%)	1.98	NS	(0.9-4.2)
**cM**						
**M0**, n (%)	634 (97.2%)	378 (99.0%)	256 (94.8%)	0.96	<0.05	(0.93-0.99)
**M1**, n (%)	18 (2.8%)	4 (1.0%)	14 (5.2%)	4.95	<0.05	(1.6-14.9)

Abbreviations: **TNM =** Tumor-Node-Metastasis; **PR =** prevalence ratio; **p** = p value; **CI95% =** confidence interval 95%; **c =** clinical; **n =** number of men; **NS =** not statistically significant

**Table 4:** D'Amico risk stratification comparison of prevalence by groups.

	Total	Group A (age 45-69)	Group B (age ≥70)	PR	p	CI95%
Low, n (prevalence)	275 (1.6%)	188 (1.3%)	87 (2.7%)	2.0	<0.01	1.6-2.6
Intermediate, n (prevalence)	256 (1.5%)	140 (1.0%)	116 (3.5%)	3.6	<0.01	2.8-4.6
High, n (prevalence)	121 (0.7%)	54 (0.4%)	67 (2.0%)	5.4	<0.01	3.8-7.7
PCa cases, n (prevalence)	652 (3.7%)	382 (2.7%)	270 (8.2%)	2.9	<0.01	(2.5-3.4)
Total, n	17,571	14,287	3,284	-	-	-

Abbreviations: **PR =** prevalence ratio; **p =** p value; **CI95% =** confidence interval 95%; **n =** number of men; **PCa =** prostate cancer

### D'Amico risk classification stratification

The stratification by the D'Amico risk classification demonstrated that considering the PCa cases, 377 of 652 men (57.8%) presented with intermediate- and high-risk disease. In the group of men aged 45-69 years, 50.8% of PCa cases were intermediate- and high-risk disease at diagnosis. In group B (aged ≥70), 67.7% of PCa cases presented with intermediate- and high-risk.

The analyses of prevalence showed that in the group of men aged ≥70, the prevalence of intermediate-risk PCa was 3.6 times higher [PR 3.6, CI95% (2.8-4.6), p <0.01], and the prevalence of high-risk disease was 5.4 times higher [PR 5.4, CI95% (3.8-7.7), p <0.01] when compared to group A, as seen in Table-4.

## DISCUSSION

Our literature review found relevant studies with concordant results. In 2015, Muralidhar et al. published a retrospective paper utilizing SEER data (n=383.039), showing positive association between advanced age and worse Gleason scores, as well as progressively greater proportions of patients with high-risk disease in older age ranges (20). One hypothesis is the drop in testosterone levels in senior men, possibly associated with more aggressive disease (21). Other plausible reason for this phenomenon is the proliferation of undifferentiated cells that represented only a small fraction of the tumoral volume at the beginning of the disease and had time to multiply over time. Other studies also reported the association of older age with more aggressive disease and worse outcomes (22). Of note, Sun et al. reported in a retrospective cohort similar findings in patients aged ≥70, while interestingly demonstrating a migration in T staging as time passed after the PSA era: in the first years of the screening, the tumoral volume, pT staging and PSA levels were higher, but there was no difference in Gleason score along the period studied, suggesting that the PSA screening impacted on the neoplastic growth without affecting its biology (23).

The median age at diagnosis found in our sample was 68 years, similar to the USA's National Cancer Institute data (66 years) (2). Nevertheless, we studied a population in a scenario of low socioeconomic status and poor access to specialized medical assistance, which is suggested by the fact that only 29% of the men had any previous PSA testing, reflecting the low frequency of PCa screening in the Brazilian population. These low rates of early diagnosis and treatment in the areas studied may have led to data reflecting the actual natural history of the disease. We also observed that only 32.6% of men aged ≥70 had at least one previous PSA measurement, so that more than two thirds of those men had not been evaluated over the age range when screening may be more beneficial. The “contamination rate” by previous PSA testing in our study was low, when compared to the literature: ERSPC reported rates around 25% (4). PLCO reported a 40% rate in the control arm at the first year (24), and recent analyses of a rate of contamination of 90% in control group participants led the PLCO authors to interpret their results as “no benefit of organized versus opportunistic screening” (5–7). It shows the difficulty in selecting homogeneous samples for prospective trials with long follow-up.

It is notable that 26.8% of men in our study were called for biopsy due to an altered DRE. This rate was superior to the 18% reported by Richie et al. in 1993, at the beginning of the PSA era (25). It may be a consequence of the low screening rate in Brazil, as by definition DRE detects tumors with volume superior to T1c cases (26), more often found in countries where early detection policies were implemented long ago.

The overall biopsy positivity rate in our study was 39.6%. It was 38% higher in the group of men aged ≥70 (PR 1.38). Although comparisons are difficult to draw, these values are superior to the 25% positivity rate found in ERSPC (4). This increase in the biopsy positivity rate associated with ageing goes along with the literature. Increasing positivity rate has been reported in elderly men, reaching 81% in men aged 80 or more (27). Our results demonstrated that men aged ≥70 had a threefold increase in PCa prevalence (PR 2.9) compared to younger men. This finding is in agreement with the well-established concept of increasing PCa prevalence associated with ageing reported in autopsy studies (28).

Our results showed that, when compared to the younger age range, PCa cases among men aged ≥70 had higher mean PSA levels and in higher ranges, with greater probability of presenting with PSA >10ng/ml (OR 2.63), which means increased chance of intermediate- and high-risk disease at diagnosis. This finding is probably linked with the markedly higher prevalence of unfavorable histological biopsy results (Gleason ≥8) in the group of elderly men (PR 3.6), which is also concordant with previous studies (29).

There was no significative difference in the T2-T4 and N1 staging between the age groups studied. However, we found that men aged ≥70 with PCa had a 5-fold chance of presenting with metastatic disease (PR 4.9). The natural history of PCa shows that 5-year survival lowers considerably in patients with M1 disease (2).

As expected, in our study the results of PSA, clinical staging and Gleason score were linked with concordant and statistically significant findings in the D'Amico risk stratification (Table-4). The prevalence of intermediate- and high-risk PCa at diagnosis was substantially higher in men aged ≥70 (PR 3.6 and 5.4, respectively), which is very relevant considering that in advanced stages progression and death due to PCa are more likely, as well as the costs of medical assistance rise and quality of life declines, so these men in theory could have benefits with screening and treatment (2, 12, 23).

Some limitations of our study must be taken into consideration. Although it demonstrated worse clinical, laboratorial and pathological features of PCa in men aged ≥70, its retrospective design does not allow recommending the systematic mass screening on this age range. Also, despite our large sample, the opportunistic screening performed may have led to a selection bias, due to the following reasons:

- The volunteer individuals enrolled were possibly more motivated to detect the disease, which may not be true to the general population. Those motivated people might be more cautious with their own health, and that may impact outcomes.
- The areas studied were poor, possibly with a frequency of the analyzed variables different from the whole Brazilian population, if we considered more privileged areas. The Brazilian Society of Urology reported a related phenomenon: poorer people treated in public Brazilian hospitals had older age, higher PSA levels and more metastatic disease at diagnosis (30).

Furthermore, the high ethnic miscegenation in Brazil is a relevant issue, considering that African-descendent populations might behave quite differently from Caucasians (2, 3), providing matter for further research.

To our knowledge, there are no Brazilian papers about PCa screening in this age range. Published literature studied mainly American and European populations. Therefore, this is the first study in Latin America assessing PCa screening in elderly men, concerning the Brazilian reality.

Considering the lack of systematic screening (which is important to emphasize that is not recommended by the government and INCA), our findings may support the concept that screening in men aged over 70 years and life expectancy of at least 10 years may be relevant in Brazil, regarding public health policies and personalized medicine.

## CONCLUSIONS

Our study demonstrated a higher prevalence of prostate cancer and a more aggressive pattern of the disease in the age group above 70 years when compared to younger men: higher PSA levels, undifferentiated Gleason score and metastatic dissemination more prevalent, as well as a higher prevalence of intermediate- and high-risk disease at diagnosis.

## ABBREVIATIONS

BCH: Barretos Cancer Hospital
c: clinical (TNM staging)
CI95%: confidence interval 95%
DRE: digital rectal examination
ERSPC: European Randomized Study of Screening for Prostate Cancer
INCA: Brazil's National Cancer Institute
M: M staging
MCPU: Mobile Cancer Prevention Units
n: number (of men)
N: N staging
NS: not statistically significant
OR: odds ratio
p: p value
PCa: prostate cancer
PLCO: Prostate, Lung, Colorectal and Ovarian cancer screening study
PR: prevalence ratio
PSA: prostate-specific antigen
SD: standard deviation
SEER: Surveillance, Epidemiology and End Results Program
T: T staging
TNM: Tumor-Node-Metastasis
TRUS: transrectal ultrasound
USA: United States of America
